# Study on the Damage of Fiber-Reinforced Seawater Sea Sand Concrete by Freezing and Thawing of Seawater

**DOI:** 10.3390/ma17081910

**Published:** 2024-04-20

**Authors:** Chuanwu Sun, Xuezhi Wang, Ming Xin, Jingjing He

**Affiliations:** 1 School of Civil and Architectural Engineering, Liaoning University of Technology, Jinzhou 121001, China; scw18914870102@163.com (C.S.); xmmyemail@163.com (M.X.); 2 Power China Northwest Engineering Corporation Limited, Xi’an 710065, China; hejing_86@126.com

**Keywords:** seawater sea sand concrete, fiber, seawater freeze–thaw coupling, microstructure, grey prediction model

## Abstract

The use of seawater and sea sand as replacements for fresh water and river sand in the preparation of seawater and sea sand concrete can effectively address issues such as high transportation costs, extended construction periods, and resource wastage. Nevertheless, in northern coastal areas, the problem of concrete durability in the complex and changing marine environment is more prominent. Research on the durability of seawater sea sand concrete is beneficial to the widening of its application range. To investigate the impact of glass fiber (GF) and polyvinyl alcohol fiber (PVA) with different blending methods on the seawater freeze–thaw resistance of seawater sea sand concrete (SSC), corresponding specimens were prepared, and seawater freeze–thaw cycling tests were conducted. By adopting the slow-freezing method and combining macro-structure and micro-morphology, the damage mechanism and the deterioration law of fiber-reinforced SSC under seawater freezing and thawing were investigated. The results indicate that, macroscopically, the incorporation of GF and PVA can effectively mitigate the damage to the matrix and reduce the effects of external erosive substances on the rate of strength loss, the rate of mass loss, and the relative dynamic elastic modulus. After 75 cycles, the SSC with a total volume doping of 0.3% and a blending ratio of 1:1 showed a 41.23% and 27.55% reduction in mass loss and strength loss, respectively, and a 29.9% improvement in relative dynamic elastic modulus compared with the basic group. Microscopic analysis reveals that the combined effect of freezing and expansion forces, the expansive substances generated by seawater intrusion into the interior of the matrix, and salt crystallization all weaken the bond between aggregate and mortar, leading to accelerated deterioration of the concrete. The incorporation of fibers enables the matrix to become denser and improves its crack-resistant properties, resulting in a better durability than that of the basic group. The damage prediction model established by the NSGM(1,N) model of gray system theory exhibits high accuracy and is suitable for long-term prediction, accurately predicting the damage of seawater sea sand concrete under seawater freeze–thaw coupling.

## 1. Introduction

Concrete is one of the most widely used materials in civil engineering [1]. With the rapid development of the civil engineering industry, the demand for non-renewable resources, such as river sand and fresh water, is increasing daily [2]. Additionally, the high consumption of natural resources is causing the gradual depletion of these resources [3,4,5], posing a significant burden on the planet that humanity relies on for its survival. The scarcity of fresh-water resources has garnered the attention of civil engineering professionals. According to relevant statistics, a significant portion of the global population faces daily water shortages [6]. Furthermore, freshwater and river sand, which are crucial for concrete composition and are non-renewable resources, have been over-extracted for a long time, have been consumed excessively, and are poorly managed, leading to a waste of freshwater resources and to bursting riverbeds, river diversion, degradation of the natural environment, and an oversupply of natural resources [7,8].

For Guangdong, Hong Kong and the Macao Bay area, the construction of coastal areas and island development has been elevated to a strategic level and the construction of related areas has become the top priority and a hotspot in the field of civil engineering. Freshwater and river sand resources are more scarce and more precious in areas such as coasts and islands [9], and increased consumption will make already insufficient resources even scarcer. If conventional concrete is used, river sand and fresh water need to be transported from inland, and their long-distance transportation poses a great challenge to the actual construction in coastal and island areas, resulting in a significant increase in cost. Seawater accounts for nearly 97% of the total water on earth, and the total amount of sea sand resources in coastal areas is nearly 68.5 × 10^10^ m^3^, indicating that coastal and island areas have abundant sea sand resources and an inexhaustible supply of seawater [10]. Some researchers have proposed the use of seawater and sea sand resources in the preparation of concrete [11,12,13], which would not only conform to the principle of achieving a shortened construction period by locally sourcing materials and saving construction costs [14], but would also save natural resources and solve the problem of ecological damage [15,16]. With a wide range of prospects for application and great advantages [17,18], this has become something of great significance for the development of the marine economy in China, as it can truly be taken from the sea and also used in it [19,20].

The mechanical properties of ordinary concrete and seawater sea sand concrete (SSC) are basically the same [21]. However, the raw material itself contains a large number of corrosive ions, leading to a corrosion of its reinforcement which in turn affects the structural performance of the overall structure of which it is a part and the loss of structural load-bearing capacity, ultimately resulting in a safety accident that would be similar to the “house of seawater sand” [22]. Currently, methods, such as rinsing with fresh water, drying through stacking, and applying rust inhibitors on the surface of steel reinforcement, are commonly employed to mitigate the impact of harmful ions. While these methods can alleviate the issue to a certain extent, they also result in the wastage of freshwater resources and an increase in time cost [23]. Concrete durability is influenced by numerous factors. In the northern coastal region of China, the cold climate and the influence of seawater tides pose specific challenges. Given the unique geographical environment, the primary causes of concrete damage in this area are erosion from seawater and the effects of freeze–thaw cycles [24,25,26]. The salts present in seawater, combined with the freeze–thaw cycle, can lead to severe deformation, cracking and spalling of concrete, significantly reducing its durability and, in turn, affecting the normal operation and service life of buildings [27,28]. To enhance the durability performance of concrete, various strategies are commonly employed, including improvements in the construction process [29,30], the incorporation of fibers [31,32], and the addition of admixtures [33,34]. In addition, concrete can be reinforced by fabric-reinforced cementitious matrix (FRCM), which has the advantages of being light weight and having excellent impact, corrosion and moisture resistance; each of which indicate it to be a better solution to the problem of reinforcing concrete in harsh environments. This reinforcement system uses a woven fiber mesh embedded in a cementitious material to form a composite reinforcement layer, where the inorganic cementitious material is bonded to the surface of the concrete and the loads acting on the structure are transferred through the inorganic cementitious material to the woven fiber mesh. Fiber is commonly used because of its low cost and ease of construction. Fibers have a significant effect on the improvement of the properties of the substrate, such as resistance to cracking, tensile bending, and compression. Fiber can withstand tensile stress on cracks, so that the surface of the specimen rupture fragments “break off but do not fall down”, meaning that the matrix “cracks but remains intact”, so that ductile damage is inflicted gradually. Low modulus of elasticity fibers (PVA, PPF) and high modulus of elasticity fibers (GF, BF) have different divisions of work inside the specimen, with the former mainly inhibiting the formation of early cracks and the latter mainly preventing the persistence of wide cracks [35]. The combination of two fibers with different properties has a growth-enhancing effect on performance. The presence of corrosive ions such as Cl and ${SO}_{4}^{2-}$ makes SSC brittle, if the fiber is mixed into SSC at a certain dosage, it can fill some of the defects inside the matrix, restrain the development of micro-cracks and crack width, and then achieve the effect of concrete toughening and strengthening.

In summary, seawater sea sand concrete (SSC) is exposed to harsh environments when used in in the northern coastal areas, and current scholars have mainly focused on the effects of single corrosive ions and single factors on concrete [36,37]. However, the study of single factors, such as the primary ions (Cl and ${SO}_{4}^{2-}$) in seawater and the freeze–thaw cycles on the matrix, is not sufficient to simulate the real situation [38]. At this stage, there is a dearth of research on the effect of real seawater erosion–thaw cycle coupling (hereinafter referred to as “coupling”) on the durability of fiber-reinforced SSC. Based on this, this paper analyzes the effect of coupling on the performance of SSC, explores the damage mechanism by observing the changes in the microstructure of SSC after coupling, and predicts the damage of SSC by using the NSGM(1,N) model, This aims to provide a certain theoretical basis for the promotion and application of SSC in the later stage.

## 2. Experimental Design

### 2.1. Raw Material

The raw materials for this test include cement, seawater, sea sand, natural aggregates, glass fibers and polyvinyl alcohol fibers.

**Figure 1:**
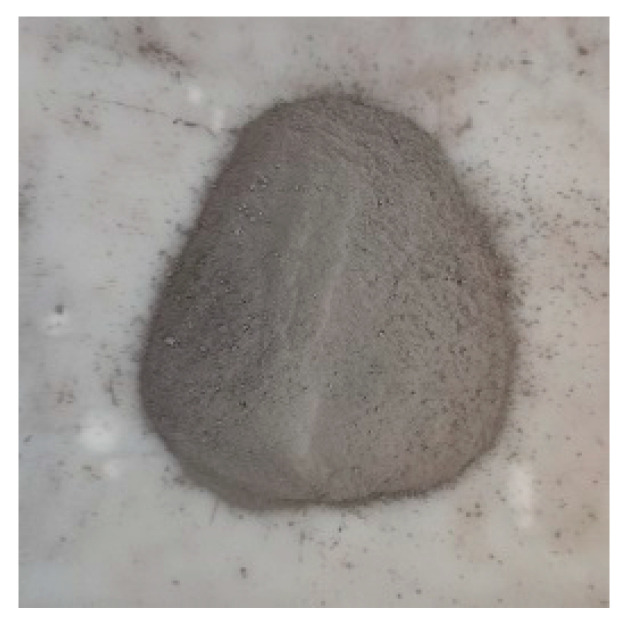
Cement.

Cement: the cement used was P-O 42.5 grade ordinary silicate cement of Jinzhou North Cement Co. (Jinzhou, China). As shown in Figure 1. The specific surface area was 320~340 m^2^/kg, its chemical composition and content are shown in Table 1, and the remaining indexes are shown in Table 2.

**Figure 2:**
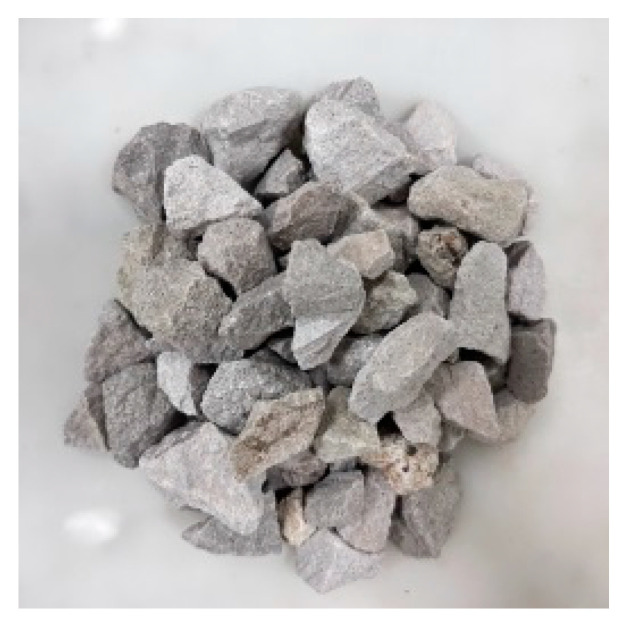
Coarse aggregate.

Coarse aggregate: local natural crushed stone from Jinzhou was used, with a grain size of 5~20 mm, the physical indexes are shown in Table 3, and the external morphology is shown in Figure 2.

**Figure 3:**
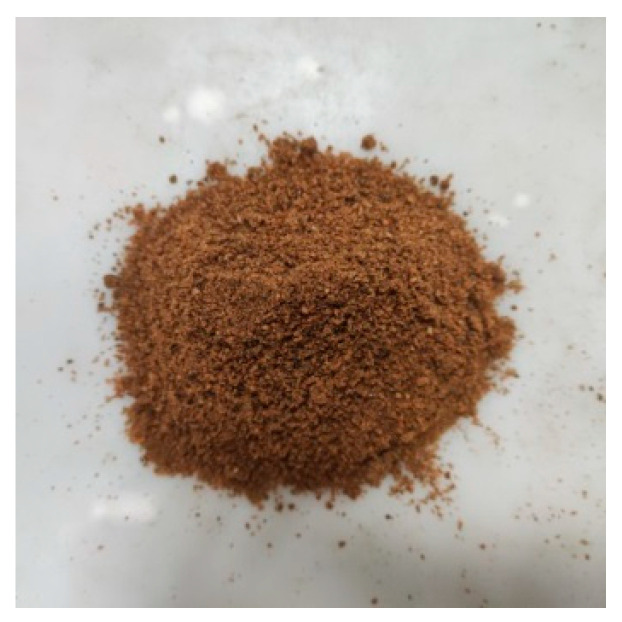
Fine aggregate.

Fine aggregate: sea sand from the waters of the Bohai Sea around Jinzhou City was used, with a fineness modulus and water content of 2.33 and 2.8, respectively, belonging to medium sand for which the chlorine ion content was 0.021%. The sea sand is shown in Figure 3.

**Figure 4:**
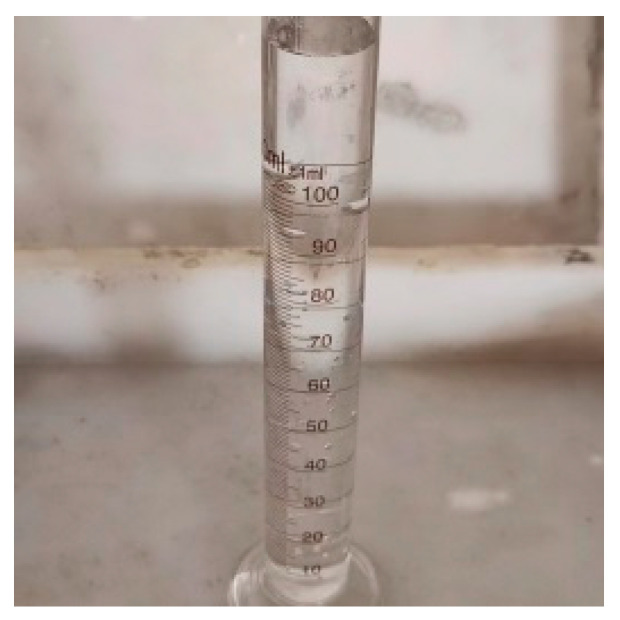
Seawater.

Seawater: the seawater was selected from the sea near Bijiashan in Jinzhou City, Liaoning Province. The specific ionic composition of the seawater is shown in Table 4, and the seawater itself is shown in Figure 4.

**Figure 5:**
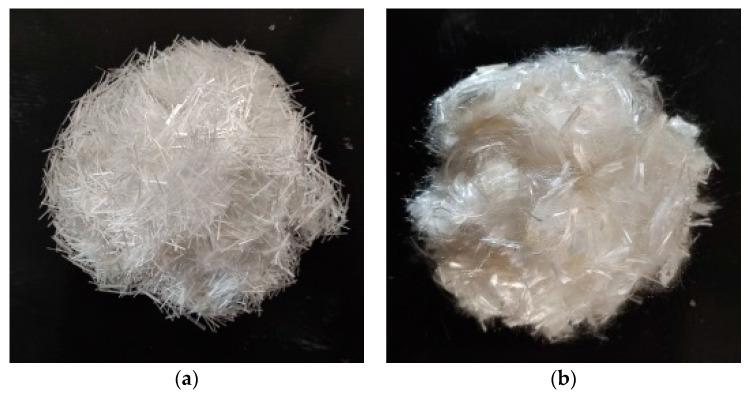
Fiber Appearance: (**a**) GF Fiber; (**b**) PVA Fiber.

Fibers: the fibers used in this test are glass fiber (GF) and polyvinyl alcohol fiber (PVA), the indexes of which are detailed in Table 5 and their appearance shown in Figure 5.

### 2.2. Mixing Ratio Design

GF and PVA fibers were mixed into C40 concrete at 0.1%, 0.2% and 0.3% by volume in a single blending, and the blended fibers were mixed into the concrete according to the total volume blending of 0.3%. The blending ratios of GF and PVA were 1:2, 1:1, and 2:1, respectively, and the tests were conducted in a total of 10 groups. The design of the mix ratio was carried out in accordance with the “Specification for the Design of Mix Ratio of Ordinary Concrete,” as shown in Table 6.

### 2.3. Instrumentation

The freeze–thaw equipment comprised a CLD automatic low-temperature freeze–thaw tester produced by Beijing Dadi Huaxin Instrument and Equipment Technology Co., Ltd. (Beijing, China); the data collection of mass loss rate was accomplished using an electronic scale produced by Yongkang Yongzhou Weighing Instrument Trading Co., Ltd. (Yongzhou, China);Loss of compressive strength data is collected using a hydraulic servo press produced by Changchun Kexin Testing Instruments Co, with the range of the press being 300 kN. (Changchun, China); Dynamic elastic modulus data acquisition was accomplished using a DT-W18 dynamic elastic modulus measuring instrument,( Beijing, China); microscopic image acquisition was accomplished using a TESCAN MIRA LMS-type scanning electron microscope (Brno, Czech Republic).

### 2.4. Specimen Preparation

**Figure 6:**
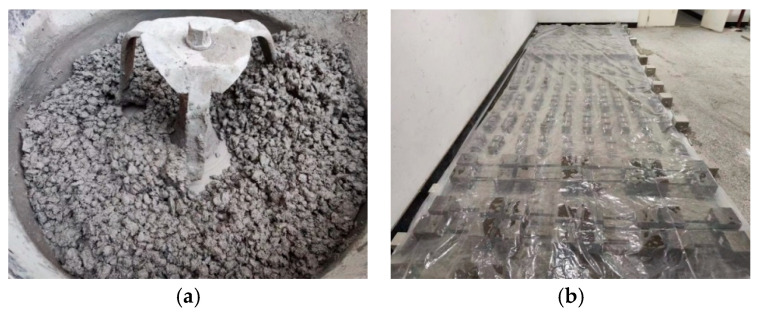
Specimen-making process. (**a**) Specimen production and (**b**) specimen maintenance.

To minimize test errors, the sea sand was adequately dried before use. During the mixing process, the sea sand, coarse aggregate and cement were first put into the mixer and fully mixed for 60S. Then, GF fiber and PVA fiber were manually added and dry mixed for about 3 min, before adding the seawater. After cement, sea sand and other materials were fully mixed, they were put into the mold and placed on a vibrating table to be fully vibrated, compacted, and smoothed. the completed specimens were then sent to the curing room in turn for numbering and standing. Twenty-four hours later, the specimen was made ready for maintenance, covered with plastic film, and sprayed with water. The test data were collected after 28 days of maintenance in a standard curing room (temperature 20 ± 3 °C, humidity greater than 95%), as shown in Figure 6.

### 2.5. Performance Test

According to the “Standard for Long-term Performance and Durability Test Methods of Ordinary Concrete” and the “Standard for Test Methods of Physical and Mechanical Properties of Concrete,” [39,40] the slow freezing method was used to study the damage destruction law of SSC by coupling action 25, 50, and 75 times, the 100 mm × 100 mm × 100 mm cube specimen was subjected to mass loss and strength loss data collection and the 100 mm × 100 mm × 400 mm prismatic specimens were subjected to dynamic elastic modulus data collection.

#### 2.5.1. Test Procedure

**Figure 7:**
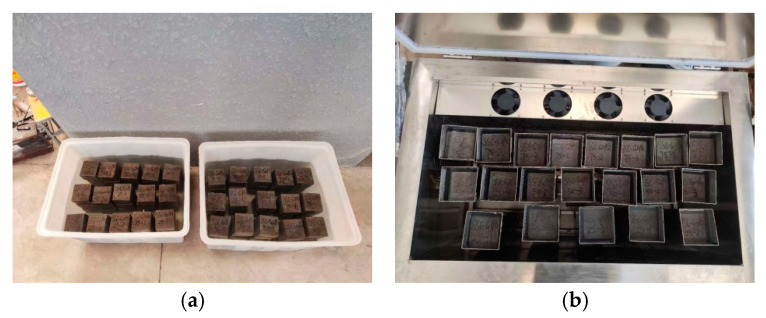
Seawater freeze–thaw coupling test. (**a**) Specimen immersion and (**b**) specimen placement.

(1) We removed the specimen from the curing room, selected the specimen with a flat surface and no missing corners, and soaked it in a white bucket filled with seawater at a temperature of 18–22 °C. The distance between the liquid surface of the bucket and the top surface of the specimen was 4 cm, and the soaking time was 4 days. This ensured that the water retention state was reached, as shown in Figure 7a.
(2) We placed the soaked specimen into a stainless-steel groove measuring 120 mm × 120 mm × 480 mm and poured the original seawater into the stainless-steel groove. During the test, we ensured that the distance between the side of the specimen and the side wall of the stainless-steel groove was 2 cm, and that the seawater exceeded the top surface of the specimen by 2 cm.
(3) We placed the stainless-steel tank containing seawater and test specimens into the freeze–thaw box, as shown in Figure 7b. We carried out 25, 50, and 75 cycles of freezing and thawing cycles of seawater. When the melting and freezing time exceeded 4 h, we poured distilled water into the external automatic water melting cycle box and maintained the melting temperature within the range of 18–20 °C, while ensuring that the temperature in the box remained within the range of −20 °C to −18 °C. We replaced the seawater solution used for soaking every other week.
(4) Once the required number of cycles had been reached, we removed the specimen from the freeze–thaw machine and noted the changes in compressive strength, mass and dynamic elastic modulus of the concrete cube before and after the freeze–thaw cycles. If the mass loss rate exceeded 5% or the strength loss rate exceeded 25%, we discarded the specimen.

#### 2.5.2. Data Acquisition

(1) The value of mass loss rate was calculated according to Formula (1).

(1) $$\Delta W_{ni}=\frac{W_{0i}-W_{ni}}{W_{0i}}\times 100$$

where $\Delta W_{ni}$ is the mass loss rate (%) of the *i*th concrete specimen after experiencing seawater erosion for N days, $W_{0i}$ is the mass (g) of the *i*th concrete specimen without experiencing seawater erosion, and $W_{ni}$ is the mass (g) of the *i*th concrete specimen after experiencing N freeze–thaw cycles.

(2) The rate of strength loss is shown in Formula (2).

(2) $$\Delta f_{c}=\frac{f_{c0}-f_{cn}}{f_{c0}}\times 100$$

where Δ*f*_c_ is the strength loss rate (%) of the *i*th concrete specimen after experiencing seawater erosion for N days, *f*_c0_ is the strength of the *i*th concrete specimen without experiencing seawater erosion (MPa), and *f_cn_* is the strength of the *i*th concrete specimen after experiencing N freeze–thaw cycles (MPa).

(3) The relative dynamic modulus of elasticity was calculated according to Formula (3).

(3) $$P_{n}=\frac{E_{n}}{E_{0}}$$

where *P_n_* is the relative dynamic modulus of elasticity, *E_n_* is the modulus of elasticity (MPa) measured in the concrete specimen after N days of seawater erosion, and *E*_0_ is the initial modulus of elasticity (MPa) of the concrete specimen.

## 3. Results and Analysis

The test results are shown in Table 7. SSC in the specimen number indicates that it is part of the general group, G is glass fiber, and P is polyvinyl alcohol fiber, e.g., G1P2 represents 0.1% and 0.2% doped GF and PVA fibers, respectively.

### 3.1. Experimental Phenomena

**Figure 8:**
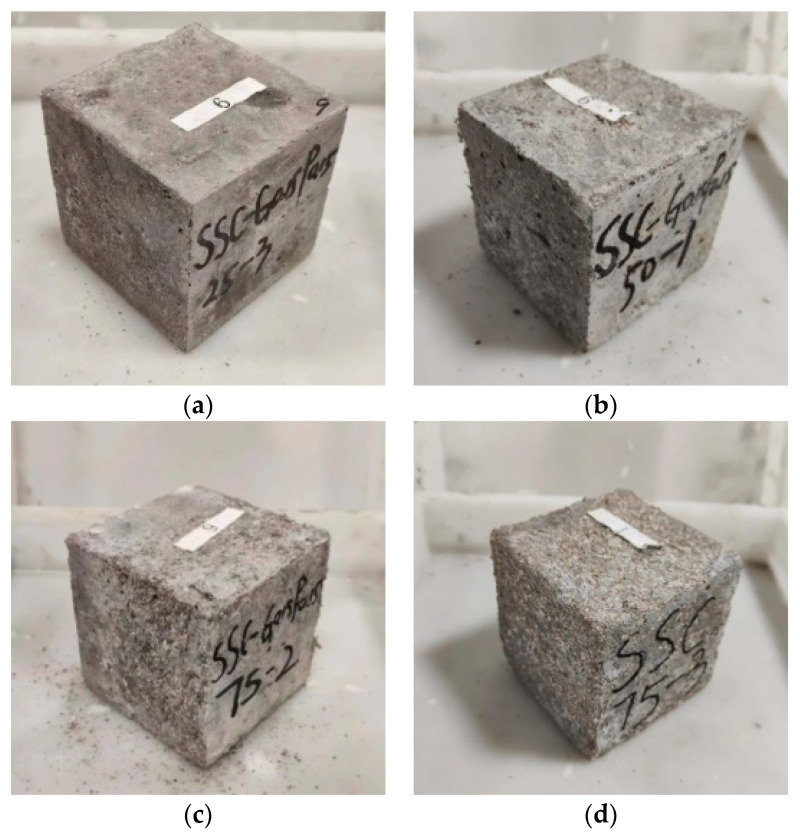
Degraded forms of the specimens. (**a**) SSC-G0.15P0.15—25 times, (**b**) SSC-G0.15P0.15—50 times, (**c**) SSC-G0.15P0.15—75 times, and (**d**) SSC—75 times.

Figure 8a–c show the damage morphologies of the outer surface of SSC-G0.15P0.15 under 25, 50 and 75 couplings. It can be observed that, with the gradual increase in the number of couplings, the honeycomb pockmarks and holes on the surface of the specimen gradually increase. After 25 coupling actions, the damage of the specimen is not obvious, a small number of small craters appear at the edges, and the surface flatness of the specimen is good, with clear edges and corners. When the number of coupling actions reaches 50, the phenomena of mortar shedding, roughness and unevenness appear on the surface of the specimen and the depth of mortar shedding is seen to be lower. The degree of mortar shedding at the edges is smaller than in the center area of the specimen, and the holes and pits on the surface are clearly increased compared with those after 25 coupling actions; however, the phenomenon of aggregate exposure did not occur. With the increase in the number of couplings to 75, seawater erosion and freezing forces damage the specimen from the surface and gradually deepen. Part of the surface of the specimen is covered with a layer of “fluffy” mortar floater, which is loose. The surface of the specimen is uneven, with deeper layers of the slurry spalling off, resulting in a small portion of the aggregate being exposed. The analyzed reasons are, firstly, that with the increase in coupling times, the gradual increase of “AFt” and “Friedel” salts within the matrix aggravates the generation of expansion stress, and the crystallization pressure generated by the salt components in the seawater destroys the bond between the aggregate and the mortar. The crystallization pressure generated by the salt components in seawater destroys the bond between the aggregate and mortar. Secondly, the synergistic working effect between the constituent materials can result in additional stresses due to the different coefficients of expansion between the components, leading to internal expansion and a large number of cracks. Thirdly, the pores of the SSC have a concentration difference due to ice crystals pressing on the unfrozen seawater in the pores, which generates osmotic pressure and results in the destruction of the pore wall and the exacerbation of slurry dislodgement. When comparing Figure 8c,d, the improvement of fiber on the damage to the specimen after freezing and thawing is clear. Serious aggregate exposure occurs on the surface of SSC, and serious corrosion damage occurs at a deeper distance from the surface of the specimen. The initial volume and the integrity of the specimen are far less than those of the doped fiber series, mainly because the doped fibers are able to effectively bear the tensile stresses generated by coupling action and thus delay the corrosion deterioration of the specimen. Observation of the specimen surface revealed a large number of salt frost crystals. The main reason for this is that the raw material of the matrix contains a large number of salts. Seawater undergoing the freeze–thaw cycle penetrates into the interior of the matrix through the tiny cracks, and when the salt concentration reaches saturation, salt crystals are precipitated.

### 3.2. Rate of Quality Loss

**Figure 9:**
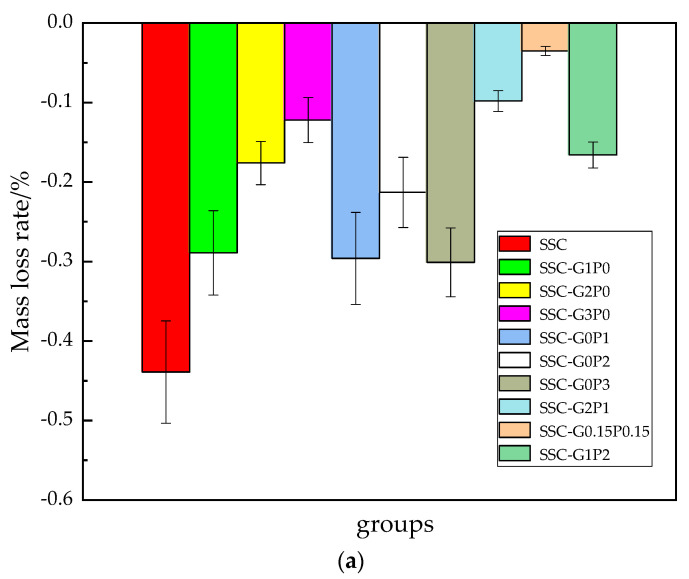

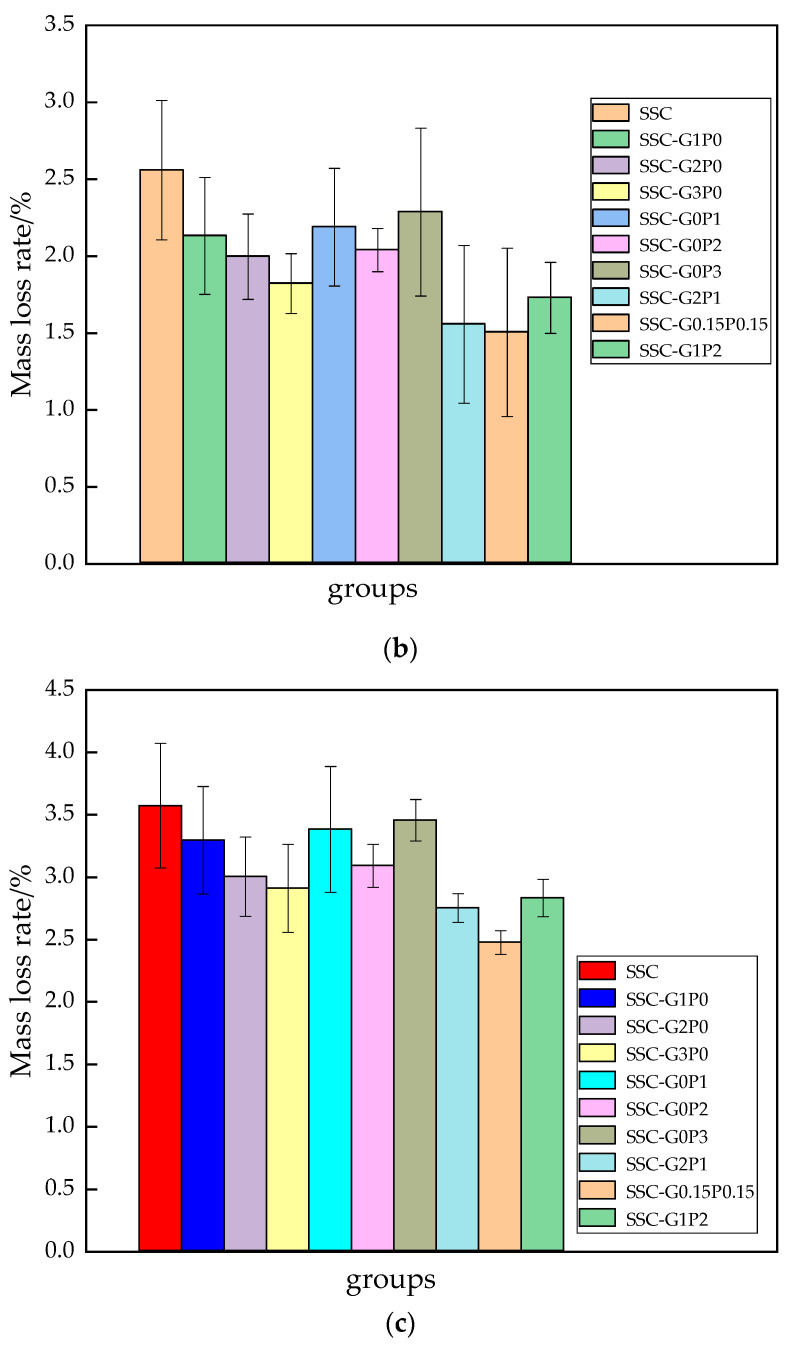
Mass loss rate of the specimen under coupling action: (**a**) 25 times, (**b**) 50 times, and (**c**) 75 times.

Figure 9 shows the changes in mass of each group after reaction coupling. It can be seen that the mass loss rate is lower than that of SSC, regardless of whether GF and PVA are blended singly or mixed. The rate of mass loss in all groups showed a decreasing and then increasing trend with the increase in the number of couplings. After 25 coupling actions, the mass of all of the groups increased compared with the pre-freeze–thaw period, with SSC-G0.15P0.15 showing the least increase in mass, −0.098%, and SSC showing the greatest increase in mass, −0.439%. The reason for this is that, at the beginning of the freeze–thaw cycle, the expansion force generated by the freeze–thaw cycle causes small cracks inside the matrix, providing a channel for seawater to invade the interior of the matrix. The seawater enters the interior of the specimen and the incompletely hydrated cementitious materials continue to undergo a hydration reaction that generates calcium substances, resulting in an increase in quality. In addition, Cl and ${SO}_{4}^{2-}$ from seawater hydrate with the cement to form “AFt” and “Friedel” salts, which fill some of the pores and increase the mass of the matrix. At 50 and 75 coupling actions, 0.3% GF doping, 0.2% PVA doping, and 1:1 mixing ratio series showed the most significant improvement in mass loss. At 50 coupling actions, the series reduced by 41.53%, 24.03% and 66.01% compared with the basic group, after 75 coupling actions, the series reduced by 28.84%, 20.32% and 41.23% compared with the basic group. Comparison of the data shows that the blended series is better than the remaining groups, which is mainly due to the existence of a good positive synergy between the GF–PVA, in which the two components of the matrix other overlap each other and become synergistic. When the external load is transferred to the fibers, the fibers can play a role in consuming the load and thus effectively preventing the generation of cracks within the slurry, as a result the blended fibers improve the frost resistance more than the remaining groups, and have a positive effect on the surface of the specimen in terms of avoiding exposure of the aggregates. With the increase in the number of coupling actions, the matrix is subjected to a variety of stresses, such as expansion stress, crystallization stress, and penetration stress, resulting in further expansion of matrix cracks, and thus an aggravation of the deterioration. In addition, Mg^2+^ in seawater reacts with C-S-H gel, displacing Ca^2+^ in the gel to generate M-S-H gel. The low cementation force of M-S-H gel makes the bonding force of the interfacial transition zone decrease, leading to the detachment of the deep slurry, which results in a rapid increase in the rate of quality loss.

### 3.3. Rate of Strength Loss

**Figure 10:**
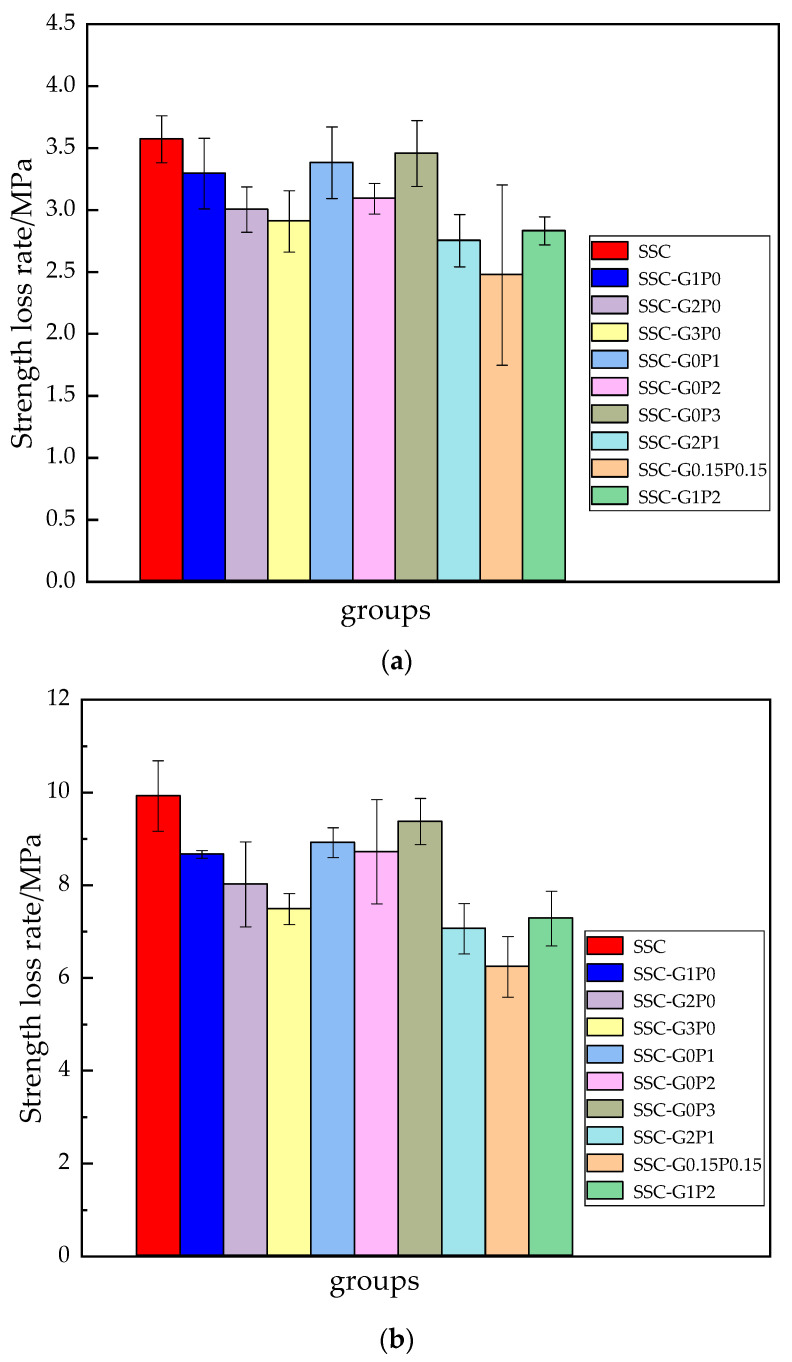

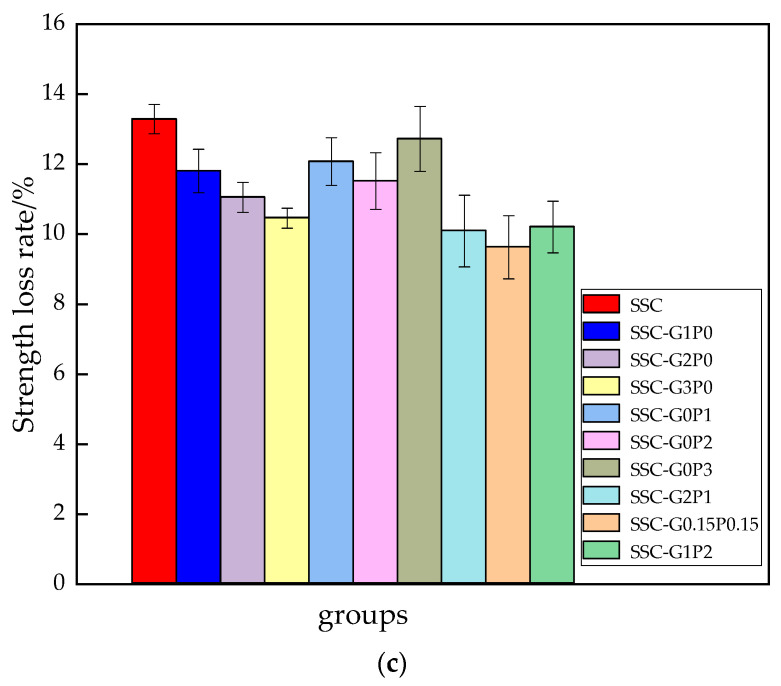
Strength loss rate of the specimens under coupling action: (**a**) 25 times, (**b**) 50 times, and (**c**) 75 times.

As can be seen from Figure 10, the strength loss rate of each group shows a gradual increase with the increase in the number of coupling actions. The main reason is that, at the early stage of freezing and thawing, the freezing and expanding force leads to the generation of small cracks on the surface of the concrete. Seawater then enters the interior of the matrix through these surface cracks, and the gel material generates the expansion of soft substances such as “AFt” and “Friedel” salt. “Friedel” salt consumes C-S-H gel, gradually reducing its gelation capacity. The expansion pressure generated inside the matrix destroys the compactness, causing internal porosity and weakening the durability of the test specimen, resulting in a greater brittleness of the matrix. In addition, with the increase in the number of cycles, the alternating cycle of negative and positive temperature, freezing and expansion force, crystallization pressure and infiltration pressure constantly consume the ultimate tensile stress of the matrix. The freezing and thawing process is, in fact, a continuous pressurization–depressurization process, resulting in the internal damage of the matrix deepening. When GF was doped alone, 0.3% fiber doping was significant in terms of improving the strength loss of the matrix, which was reduced by 18.56%, 24.59% and 21.31% compared with the respective basic groups. The rate of strength loss at 0.2% PVA doping was less than the other groups when PVA was doped alone, with reductions of 13.44%, 12.14% and 13.34% over the respective basic groups. Analysis of the data shows that the fiber improves the frost resistance of SSC significantly. The reason for this is that, due to the presence of corrosive ions inside the specimen, a large number of expansive substances are produced, resulting in numerous cracks within the cement matrix. GF fiber due to the advantages of high modulus and high strength, play a certain role in reinforcing. When subjected to imposed loads, GF fiber and cementitious materials occur in the process of debonding or resistance to freezing and expansion of the force consumes a lot of energy, so that the tensile strength of the composite material has a certain enhancement of the role of the specimen toughness and tensile properties have been greatly improved. On the other hand, the excellent hydrophilicity of PVA fibers enables the cement mortar attached to the fiber surface to be fully hydrated, and a large number of C-S-H gels are attached to the interfacial transition zone (ITZ) between the fibers and other materials in the matrix, thus improving the interfacial properties between the fibers and the matrix. However, the improvement of freezing resistance of PVA is far less than that of GF, the reason for which is that GF has a better dispersion and, at an appropriate dosage, the most uniform division inside the matrix. The tensile strength of GF is far greater than that of PVA, with the attributes of high modulus of elasticity, high ultimate tensile strength and the ability to prevent the emergence of large cracks inside the matrix, which effectively enhances the matrix’s performance when resisting freezing and expansion forces. In the blended series, the 1:1 blend showed 30.68%, 37.12% and 27.55% reductions in strength loss over the respective basic groups. The mixed series is better than the single mixed series because the two fibers in the concrete are mixed together to form a fiber network structure, and the cement–aggregate connection is close, presenting a chaotic and disorderly distribution. PVA fiber has the characteristic of softness so that the specimen’s tiny pores can be filled to a certain extent. When the specimen is subjected to freezing and expansion forces, the PVA fiber is the first to produce effects that prevent and slow the development of small cracks when it reaches the limit of the specimen. When the limit load is reached, larger macro cracks appear inside the specimen and the higher tensile strength of the GF fiber continues to produce such effects. The two fibers consume part of the energy in the processes of pullout and pullout failure, thus delaying the cracking time of the specimen. In addition, the three-dimensional fiber skeleton support structure formed inside the cement matrix, fiber two by two overlap, filling the holes, the defects inside the specimen is effectively improved, the integrity of the specimen and the densification of the specimen has been significantly improved, to prevent premature failure of the specimen due to defects generated by the stress concentration when subjected to load. However, the 1:2 mixing ratio of SSC frost resistance is not as good as that of other mixing series, the reason for which is that the PVA is bundled and dispersed far less than the GF fiber. Too high a mixing amount in the concrete internal part of the agglomeration, as well as the uneven distribution phenomenon, hinders the hydration of cementitious materials, resulting in a degree of encapsulation between the fiber and the concrete and other materials, reducing the degree of denseness. When bearing external stresses, the phenomenon of uneven force and load eccentricity is likely to occur, thus the lifting effect is inferior to the rest of the groups.

### 3.4. Relative Modulus of Elasticity

**Figure 11:**
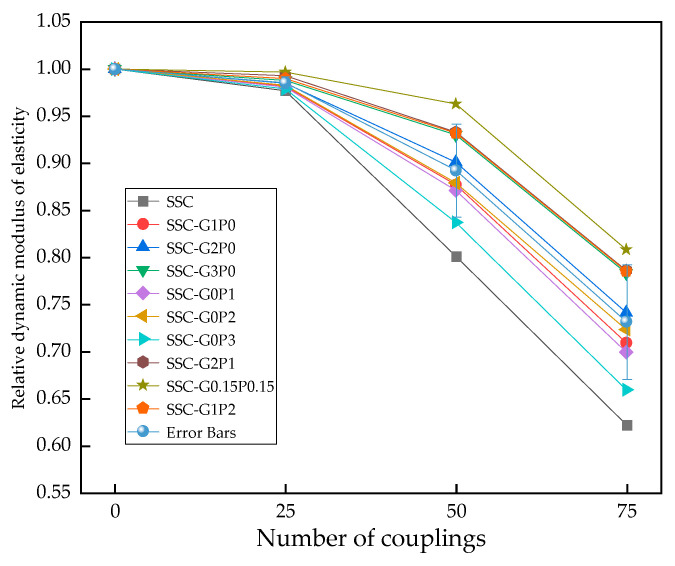
Relative dynamic elastic modulus of the specimen under coupling action.

Figure 11 responds to the change rule of the relative dynamic elastic modulus of SSC. From the figure, it can be seen that the specimens with different fiber doping and with the coupling effect gradually increased, while the relative dynamic elastic modulus shows a decreasing trend. The reason for this is that the SSC itself contains a large number of erosion ions in the raw materials, while the external erosion of substances, along with the increase in the number of coupling actions, gradually leads to the erosion of the interior of the matrix. The two together produce a large number of salt crystals and an expansion of soft substances (Aft, gypsum, etc.) and, in the transition zone of the aggregates and cementitious materials (ITZ) buildup, reduce the transition zone of the bonding so as to impede the hydration of the cementitious materials, in turn resulting in the interior of the matrix becoming brittle and porous. In addition, due to crystalline expansion caused by the crystallization of frozen water and salts in the pores, osmotic pressure develops between the supercooled water, and the specimen progressively deteriorates from the surface to the interior, resulting in a rapid decrease in the relative modulus of elasticity. Comparing the doped fiber series, the relative modulus of elasticity of the specimens was most significantly improved by GF single doping of 0.3%, PVA single doping of 0.2%, and mixing ratio of 1:1, especially in the mixing ratio of 1:1 series, which was elevated by 2.05%, 20.22%, and 29.9% compared with those of the respective basic groups. Adding fibers to the specimen with an enhanced relative modulus of elasticity plays a role in promoting our analysis, the reason for which is that the fiber in the matrix shows an internal irregular distribution, the fibers are entangled with each other, overlap, and form a three-dimensional “fiber cage” support structure, which increases its load-bearing capacity and effectiveness. In concrete, the differences in aggregate grading lead to the appearance of capillary channels or pores, meaning that the fiber can be mixed to fill part of these pores. This increases its compactness, alleviates the damage caused by freezing and expansion forces to the interior of the matrix, inhibits the development of harmful cracks, and increases its frost resistance and working efficiency.

## 4. Microanalysis

**Figure 12:**
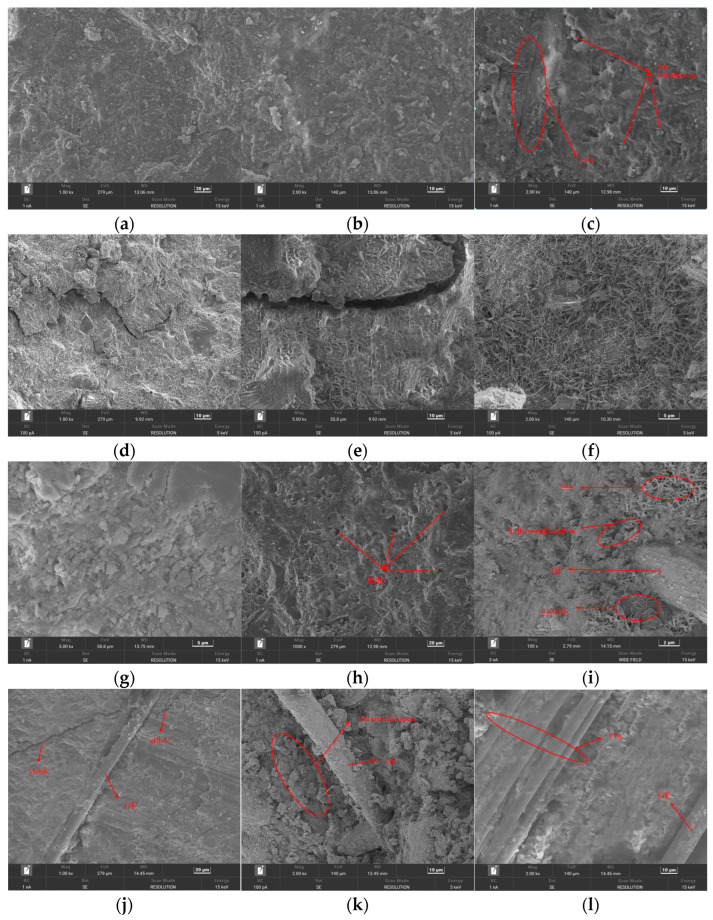
Microstructure. (**a**) SSC—0 times; (**b**) SSC-G0.15P0.15—0 times; (**c**) SSC—25 times, cracks; (**d**) SSC—50 times, cracks; (**e**) SSC—75 times, cracks; (**f**) SSC—75 times, calcovanadiumadium; (**g**) SSC—75 times, salt crystallization; (**h**) SSC—75 times, hole; (**i**) SSC-G0.15P0.15—0 times, fiber-to-matrix connection; (**j**) SSC-G0.15P0.15—75 times, fiber inhibits cracking; (**k**) SSC-G0.15P0.15—75 times, fiber-filled pores; and (**l**) SSC-G0.15P0.15—75 times, fiber synergy.

The evolution pattern of fiber-reinforced SSC corrosion and the damage pattern of specimens were investigated by SEM scanning electron microscopy of SSC and SSC-G0.15P0.15 at different coupling times. Figure 12a,b show the SEM images of SSC and SSC-G0.15P0.15 before damage, and it can be seen that, before the test, the various hydration products inside the matrix are connected with each other and have good integrity, uniform distribution, and continuity; however, it contains corrosive ions, leading to a small number of internal holes. From Figure 12c–e, it can be seen that the cracks inside the matrix gradually deepen with the increase in the number of couplings. This is mainly due to the following reasons. Firstly, microcracks inside the matrix gradually increase in freezing and thawing environments, which facilitate the intrusion of seawater and accelerate the degree of corrosion. Secondly, the interior of the matrix is constantly subjected to the freeze–thaw process, and the expansion forces increase the growth rate of the cracks, causing damage to the internal structure of the specimen. Thirdly, when the temperature is negative, some of the water in the pores freezes and forms a concentration difference with the unfrozen water in the pores, which then leads to the generation of osmotic pressure, resulting in the cracking of the concrete. The intrusion of seawater into the interior of the matrix forms a large number of salt crystals at the pores (Figure 12c), which better explains the increase in mass of the specimen after 25 coupling actions. After 75 cycles, the main corrosive substances (AFt, “Friedel” salt) are heavily enriched, and the phenomenon of “clusters” occurs, with a large number of aggregates and continuous growth in cracks and holes. The joint action of Cl^−^ and ${SO}_{4}^{2-}$ leads to the decomposition of C-S-H gel, consuming a large amount of Ca(OH)_2_. AFm, Ca(OH)_2_, C-S-H, and a small amount of AFt are cement hydration products. Seawater and sea sand added to the concrete contain the main corrosive ions that have an erosive effect on the matrix: Cl^−^, ${SO}_{4}^{2-}$, and Mg^2^⁺. Among these, seawater and sea sand react with Cl^−^ and monosulfur-type hydrated calcium aluminate (AFm), tricalcium aluminate (C_3_A), and Ca(OH)_2_ to generate “Friedel” salt, the main source of AFt. The reaction of ${SO}_{4}^{2-}$ and AFm also produces AFt. The generated AFt (needle-like rods) and “Friedel” salt (granular) are very easy to expand, resulting in a sharp increase in expansion pressure and crystallization pressure, reducing the strength and performance of the matrix. The presence of corrosives made the specimens become more brittle and deteriorated their internal integrity, in turn affecting their durability, as shown in Figure 12f,g. In addition, the generated corrosive material caused the slurry to be loose and porous, and the number of large pores increased, weakening the bond between the aggregate and the slurry. At this time, the internal densification of the matrix was greatly reduced, so that the relative modulus of elasticity appeared to decrease significantly, as shown in Figure 12h. Figure 12i shows the SSC-G0.15P0.15 specimen after zero coupling actions. As can be seen from the figure, the fibers are surrounded by AFt (needle-like rods), “Friedel” salt (granular), Ca(OH)_2_ (flaky), and C-S-H gel (flocculent). The fibers are densely bonded with the mortar, improving the internal space structure of the matrix. After being subjected to 75 cycles of freezing and thawing, the fibers effectively improved the tensile force between cracks (Figure 12j). Additionally, the fibers had a better restraining effect on the widening of the cracks, effectively enhancing the crack-resistant performance of the matrix and delaying the damage to the specimen caused by expansion stress, crystallization pressure, and freezing and expansion force. Furthermore, a large number of crystals accumulated around the fibers, and the fiber incorporation effectively filled the pores, strengthening the integrity of the specimen (Figure 12k). Incorporating both types of fibers inside the matrix (Figure 12l) creates a reliable bond with the slurry, counteracting some of the expansion stresses and crystallization pressures and reducing the impact of internal defects on performance. While PVA inhibits the development of preexisting microcracks, GF prevents the generation of macrocracks. The two complement each other’s strengths, significantly improving SSC’s resistance to erosion and frost.

## 5. NSGM(1,N)-Based Damage Prediction Models

For the characteristics of the collected test data with complexity, variability and a large discrete type, the gray system theory can make accurate predictions [41]. Zeng Bo found that the NSGM(1,N) model may be a better solution to the problem of large errors in prediction results caused by defects in parameters and other defects of the GM(1,N) model [42]. Based on this, the NSGM(1,3) was used in this test to model damage prediction and evaluate the strength loss rate and relative dynamic elastic modulus of SSC. Both are modeled in the same way, and this paper takes the modeling step of the strength loss rate after 25 coupling actions as an example.

### 5.1. Model Building

In step 1, we selected the initial seven groups of strength loss rate based on the sample data series. The last three groups of strength loss rate were predicted in order to ensure model accuracy. The dependent variable in the system characteristic series, $X_{1}^{\left(0\right)}$, represents the intensity loss rate after 25 coupling actions. The independent variable data series (related factors) for GF and PVA doping are represented by $X_{i}^{\left(0\right)}$ (i = 2,3). Equations (4)–(6) show $X_{1}^{\left(0\right)}$, $X_{2}^{\left(0\right)}$, and $X_{3}^{\left(0\right)}$.

(4) $$\begin{array}{l} X_{1}^{\left(0\right)}=(X_{1}^{\left(0\right)}(1),X_{1}^{\left(0\right)}(2),\ldots ,X_{1}^{\left(0\right)}(10))\\ =(3.572,3.382,3.092,3.457,3.295,2.832,3.004,2.753,2.909,2.476) \end{array}$$

(5) $$\begin{array}{l} X_{2}^{\left(0\right)}=(X_{2}^{\left(0\right)}(1),X_{2}^{\left(0\right)}(2),\ldots ,X_{2}^{\left(0\right)}(10))\\ =(0,0,0,0,0.001,0.001,0.002,0.002,0.003,0.0015) \end{array}$$

(6) $$\begin{array}{l} X_{3}^{\left(0\right)}=(X_{3}^{\left(0\right)}(1),X_{3}^{\left(0\right)}(2),\ldots ,X_{3}^{\left(0\right)}(10))\\ =(0,0.001,0.002,0.003,0,0.002,0,0.001,0,0.0015) \end{array}$$

In step 2, the independent and dependent 1-AGO cumulative sequences, $X_{i}^{\left(1\right)}$, are given by Equations (7)–(9).

(7) $$\begin{array}{l} X_{1}^{1}=(X_{1}^{\left(1\right)}(1),X_{1}^{\left(1\right)}(2),\ldots ,X_{1}^{\left(1\right)}(10))\\ =(3.572,6.954,10.046,13.503,16.798,19.630,22.634,25.387,28.296,30.772) \end{array}$$

(8) $$\begin{array}{l} X_{2}^{1}=(X_{2}^{\left(1\right)}(1),X_{2}^{\left(1\right)}(2),\ldots ,X_{2}^{\left(1\right)}(10))\\ =(0,0,0,0,0.001,0.002,0.004,0.006,0.009,0.0105) \end{array}$$

(9) $$\begin{array}{l} X_{3}^{1}=(X_{3}^{\left(1\right)}(1),X_{3}^{\left(1\right)}(2),\ldots ,X_{3}^{\left(1\right)}(10))\\ =(0,0.001,0.003,0.006,0.006,0.008,0.008,0.009,0.009,0.0105) \end{array}$$

Among others: $X_{j}^{\left(1\right)}(k)=\sum_{g=1}^{k}X_{j}^{\left(0\right)}(g),k=1,2,3,\cdots .$

In step 3, we calculated the mean of the immediate neighborhood series $Z_{1}^{\left(1\right)}$ for $X_{1}^{\left(1\right)}$, as shown in Equation (10).

(10) $$\begin{array}{l} Z_{1}^{\left(1\right)}=(Z_{1}^{\left(1\right)}(2),Z_{1}^{\left(1\right)}(3),\ldots ,Z_{1}^{\left(1\right)}(9))\\ =(3.477,3.237,3.275,3.376,3.064,2.918,2.879,2.831,2.693) \end{array}$$

Among others: $Z_{1}^{\left(1\right)}(k)=\frac{1}{2}\left[\left(X_{1}^{\left(1\right)}(k)+X_{1}^{\left(1\right)}(k-1\right)\right],k=2,3,\ldots ,m$

In Step 4, we constructed parameter matrices B and Y, as shown in Equations (11) and (12). Then, we calculated the development coefficient a, driving coefficient b, and amount of gray role h, as shown in Equation (13).

(11) $$B=\left[\begin{array}{ccccc} x_{2}^{\left(1\right)}(2) & x_{3}^{\left(1\right)}(2) & -z_{1}^{\left(1\right)}(2) & 1 & 1\\ x_{2}^{\left(1\right)}(3) & x_{3}^{\left(1\right)}(3) & -z_{1}^{\left(1\right)}(3) & 2 & 1\\ \vdots & \vdots & \vdots & \vdots & \vdots \\ x_{2}^{\left(1\right)}(6) & x_{3}^{\left(1\right)}(6) & -z_{1}^{\left(1\right)}(6) & 6 & 1 \end{array}\right]=\left[\begin{array}{ccccc} 0 & 0.001 & -5.263 & 1 & 1\\ 0 & 0.003 & -8.5 & 2 & 1\\ \vdots & \vdots & \vdots & \vdots & \vdots \\ 0.004 & 0.008 & -21.132 & 6 & 1 \end{array}\right]$$

(12) $$Y=\left(\begin{array}{c} x_{1}^{\left(0\right)}(2)\\ x_{1}^{\left(0\right)}(3)\\ \vdots \\ x_{1}^{\left(0\right)}(6) \end{array}\right)=\left[\begin{array}{c} 3.382\\ 3.092\\ \vdots \\ 3.004 \end{array}\right]$$

(13) $$\hat{p}={\left(B^{T}B\right)}^{-1}B^{T}Y={\left(29.5402,65.4565,-0.17329,-0.75106,3.11063\right)}^{T}$$

In step 5, the intermediate variables $u_{1}$, $u_{2}$, $u_{3}$, and $u_{4}$ were calculated from the computational parameters using Equation (14).

(14) $$u_{1}=\frac{1}{1+0.5a}=1.0949,u_{2}=\frac{1-0.5a}{1+0.5a}=1.1897,u_{3}=\frac{h_{1}}{1+0.5a}=-0.8223,u_{4}=\frac{h_{2}-h_{1}}{1+0.5a}=4.228$$

In step 6, the prediction model was derived as shown in Equation (15), and the prediction model formulas for different numbers of couplings are shown in Table 8 and Table 9.

(15) $$\begin{array}{l} x_{1}^{\left(0\right)}(k)=u_{1}(u_{2}-1)\sum_{t=1}^{k-2}\left[\sum_{i=2}^{N}u_{2}^{t-1}b_{i}x_{i}^{\left(1\right)}(k-t)\right]+\\ u_{1}\sum_{i=2}^{N}b_{i}x_{i}^{\left(1\right)}(k)+\sum_{j=0}^{k-3}u_{2}^{j}u_{3}+(u_{2}-1)u_{2}^{k-2}x_{1}^{\left(1\right)}(1)+u_{2}^{k-2}(2u_{3}+u_{4}),k=2,3,\cdots \end{array}$$

For Step 7, the residual, relative error, and average relative error formulas are shown in Equations (16)–(18)

Residuals:

(16) $$\varepsilon_{s}(k)=X_{1}^{\left(0\right)}(k)-{\hat{X}}_{1}^{\left(0\right)}(k-1),k=2,3,\ldots$$

Relative error:

(17) $$\Delta_{s}(k)=\left|\frac{\varepsilon_{s}(k)}{X_{1}^{\left(0\right)}(k)}\times 100\%\right|,k=2,3,\ldots$$

Average relative error:

(18) $$\bar{\Delta_{s}}=\frac{1}{n}\sum_{k-1}^{n}\Delta_{s}(k)$$

### 5.2. Accuracy Prediction

**Figure 13:**
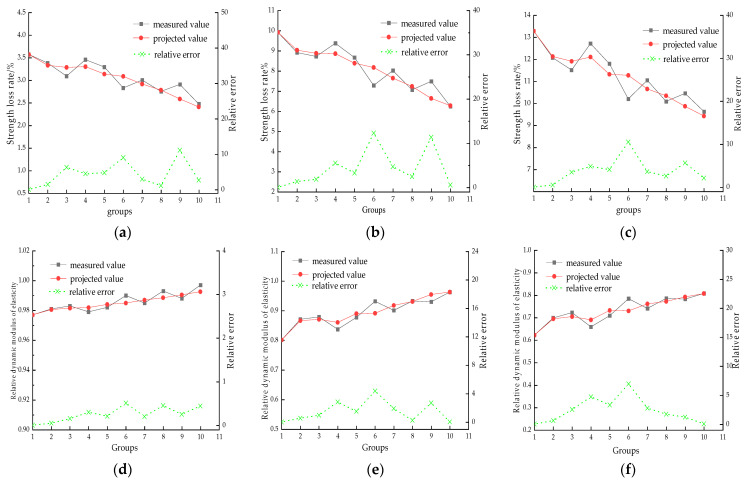
Comparison of measured and predicted values: (**a**) 25 times—strength loss rate, (**b**) 50 times—strength loss rate, (**c**) 75 times—strength loss rate, (**d**) 25 times—relative dynamic modulus of elasticity, (**e**) 50 times—relative dynamic modulus of elasticity, (**f**) 75 times—relative dynamic modulus of elasticity.

As can be seen from Figure 13, the predicted data of NSGM(1,3) under each coupling number is basically similar to the measured data, and it can be considered that the correlation between the two is strong and the credibility is high. Checking the related gray model accuracy judgment standards, the average relative error of level I prediction accuracy is <0.01, and the average relative error of level II prediction accuracy is <0.05 [43]. The average relative error is calculated according to Equations (12)–(14), and the model parameters are shown in Table 10. The average relative errors of the models established based on the strength loss rate and the relative kinetic elastic modulus are within 4.95% and 0.99%, respectively. Additionally, the prediction model of the strength loss rate is within the class II error, and the prediction model of the relative kinetic elastic modulus is within the class I error, so it can be seen that the accuracy of the prediction model based on the NSGM(1,3) is higher, and that SSC damage when undergoing coupling is applicable to the NSGM(1,N) model. This may provide new ideas for future research and promotion related to SSC-doped fibers. Furthermore, Deng Julong has indicated their belief that the model is meaningful when the development coefficient a is within (−2,2) [43]; Liu Sifeng has expressed belief that it is applicable to long-term forecasting when a ≥ −0.3 and to short-term forecasting when −0.5 ≤ a ≤ −0.3 [44]. From Table 10, it can be seen that a∈(−0.213,−0.023), meaning that the long-term damage life of fiber-reinforced seawater sea sand concrete undergoing seawater freezing and thawing cyclic coupling action can be predicted with the NSGM(1,N) damage prediction model.

## 6. Conclusions

(1) The SSC specimen without fiber mixing, under the joint action of freezing expansion force and erosive material, appeared to have a more seriously honeycombed and pockmarked surface. Additionally, the specimen structure was found to be loose, with poor resistance to frost and erosion. The fiber mixture played the role of crack blocking and toughening after the coupling effect did not appear to expose the aggregate, with both integrity and resistance to frost and erosion being greatly improved.

(2) GF and PVA fibers are mixed into the concrete to improve its performance significantly. The optimal fiber single mixing dosage of GF and PVA is 0.3% and 0.2%, respectively. In particular, a total volume mixing dosage of 0.3%, and a mixing ratio of 1:1, offered the most obvious improvement in the quality of the loss of concrete and the relative modulus of elasticity, which is better than the rest of the groups in terms of the mechanical properties of the concrete. Overall, the blended GF–PVA fibers improved the concrete properties better than the single blend of GF or PVA. After 75 freeze–thaw cycles, the mass loss rate and strength loss rate increased by 41.23% and 27.55%, respectively, compared with the basic group, and the relative elastic modulus increased by 29.9%.

(3) Through microanalysis, it can be seen that the tiny cracks formed by freeze–thaw cycles provide conditions for seawater intrusion, and that, with the increase in the number of couplings, the freezing and expansion force increases the width of the cracks in the interface zone (ITZ) of the aggregate and mortar. The salt crystals and corrosive substances such as AFt are constantly expanding in the ITZ, and the flocculent C-S-H gel is gradually disintegrated, which leads to the structure becoming looser and more porous, accelerating the degradation of the concrete. Additionally, the incorporation of fibers is able to effectively improve the anti-cracking performance of the matrix, delay the generation of cracks, and improve the durability of the specimen under coupling.

(4) The basic idea of gray system theory is introduced into the study of SSC erosion resistance, and the average relative errors of the strength loss rate damage prediction model and the relative kinetic elastic modulus damage prediction model based on the NSGM (1,3) model are within 4.95% and 0.99%, respectively. Additionally, the model accuracies are within the class II and class I errors and the relative kinetic elastic modulus damage prediction model accuracies are better than the strength loss rate damage prediction model accuracy; moreover, the development coefficient a is between −0.213 and −0.023, which indicates that the prediction model meets the long-term prediction. The relative dynamic elastic modulus damage prediction model accuracy is better than that of the strength loss rate damage prediction model. Additionally, in the grey system theory, the development coefficient a is an important index by which to evaluate whether the data can be predicted in the long run. The development coefficient a ranges from −0.213 to −0.023, and the fit between the measured value and the calculated prediction value is high, which indicates that the prediction model is in line with the long-term prediction.

## Figures and Tables

**Table 1:** Chemical composition and content of cement.

Chemical Composition	SiO_2_	Al_2_O_3_	Fe_2_O_3_	CaO	K_2_O	MgO
Quantity contained (%)	19.1	6.2	3.31	64.6	5.91	2.93

**Table 2:** Cement indicators.

Cement	Densities (g·cm^−3^)	Incipient Condensation Time (min)	Final Setting Time (min)	Flexural Strength (MPa)		Compressive Strength (MPa)	
				3 d	28 d	3 d	28 d
P-O42.5	3.1	160.5	260.5	5.18	8.88	24.59	49.87

**Table 3:** Index of the coarse aggregate.

Aggregate	Apparent Density (g·cm^−3^)	Packing Density (g·cm^−3^)	Mud Content (%)	Grain Size Grading (mm)	Needle and Flake Content (%)
Coarse aggregate	2658	1445	0.29	5–20	15

**Table 4:** Main components of seawater (mg·L^−1^).

Ion Type	${SO}_{4}^{2-}$	CI^−^	Ca^2+^	Mg^2+^	K^+^	Na^2+^
Ionic concentration	2116	15,723	257	7699	231	958

**Table 5:** Fiber index.

Fiber Type	Lengths (mm)	Densities (g·cm^−3^)	Modulus of Elasticity (GPa)	Ultimate Elongation (%)	Tensile Strength (MPa)	Geometry
GF	12	2.65	80	2.47	2300	Slub yarn
PVA	12	1.29	40	-	1750	Fasciculated

**Table 6:** Seawater sand concrete mix ratio.

Coarse Aggregate (kg·m^−3^)	Fine Aggregate (kg·m^−3^)	Cement (kg·m^−3^)	Sea Water (kg·m^−3^)	Water–Cement Ratio	Sand Rate (%)
1173	660	417	200	0.48	36

**Table 7:** Test results.

Test Grouping	Fiber Doping/%		Quality Loss Rate/%			Strength Loss Rate/%			Relative Modulus of Elasticity		
	GF	PVA	25 Times	50 Times	75 Times	25 Times	50 Times	75 Times	25 Times	50 Times	75 Times
SSC	0	0	−0.439	1.315	2.559	3.572	9.926	13.287	0.977	0.801	0.622
SSC-G1P0	0.1	0	−0.289	1.107	2.131	3.295	8.667	11.802	0.982	0.877	0.709
SSC-G2P0	0.2	0	−0.176	0.942	1.996	3.004	8.02	11.051	0.985	0.901	0.741
SSC-G3P0	0.3	0	−0.122	0.769	1.821	2.909	7.485	10.456	0.988	0.930	0.783
SSC-G0P1	0	0.1	−0.296	1.121	2.188	3.382	8.917	12.073	0.981	0.871	0.699
SSC-G0P2	0	0.2	−0.213	0.999	2.039	3.092	8.721	11.515	0.983	0.879	0.723
SSC-G0P3	0	0.3	−0.301	1.243	2.286	3.457	9.374	12.718	0.979	0.837	0.659
SSC-G2P1	0.2	0.1	−0.098	0.695	1.556	2.753	7.064	10.089	0.993	0.933	0.786
SSC-G0.15P0.15	0.15	0.15	−0.035	0.447	1.504	2.476	6.241	9.626	0.997	0.963	0.808
SSC-G1P2	0.1	0.2	−0.166	0.718	1.728	2.832	7.282	10.203	0.990	0.932	0.785

**Table 8:** Prediction model of strength loss rate.

Number of Seawater Freeze–Thaw Couplings	Predictive Model
25	$\begin{array}{l} {\hat{x}}_{1}^{\left(0\right)}(k)=0.2077\sum_{t=1}^{k-2}\left[\sum_{i=2}^{4}1.1897^{t-1}b_{i}x_{i}^{\left(1\right)}(k-t)\right]+\\ 1.0949\sum_{i=2}^{4}b_{i}x_{i}^{\left(1\right)}(k)-0.8223\sum_{j=0}^{k-3}1.1897^{j}+0.1897\times 1.1897^{k-2}x_{1}^{\left(0\right)}(1)+\\ 2.5834\times 1.1897^{k-2},k=2,3,\cdots \end{array}$
50	$\begin{array}{l} {\hat{x}}_{1}^{\left(0\right)}(k)=0.0379\sum_{t=1}^{k-2}\left[\sum_{i=2}^{4}1.0372^{t-1}b_{i}x_{i}^{\left(1\right)}(k-t)\right]+\\ 1.0186\sum_{i=2}^{4}b_{i}x_{i}^{\left(1\right)}(k)-0.7710\sum_{j=0}^{k-3}1.0372^{j}+0.0372\times 1.0372^{k-2}x_{1}^{\left(0\right)}(1)+\\ 8.5245\times 1.0372^{k-2},k=2,3,\cdots \end{array}$
75	$\begin{array}{l} {\hat{x}}_{1}^{\left(0\right)}(k)=0.2660\sum_{t=1}^{k-2}\left[\sum_{i=2}^{4}1.2377^{t-1}b_{i}x_{i}^{\left(1\right)}(k-t)\right]+\\ 1.1189\sum_{i=2}^{4}b_{i}x_{i}^{\left(1\right)}(k)-4.0213\sum_{j=0}^{k-3}1.2377^{j}+0.2377\times 1.2377^{k-2}x_{1}^{\left(0\right)}(1)+\\ 8.5118\times 1.2377^{k-2},k=2,3,\cdots \end{array}$

**Table 9:** Prediction model of relative dynamic elastic modulus.

Number of Seawater Freeze–Thaw Couplings	Predictive Model
25	$\begin{array}{l} {\hat{x}}_{1}^{\left(0\right)}(k)=0.1945\sum_{t=1}^{k-2}\left[\sum_{i=2}^{4}1.1786^{t-1}b_{i}x_{i}^{\left(1\right)}(k-t)\right]+\\ 1.0893\sum_{i=2}^{4}b_{i}x_{i}^{\left(1\right)}(k)-0.1725\sum_{j=0}^{k-3}1.1786^{j}+0.1786\times 1.1786^{k-2}x_{1}^{\left(0\right)}(1)+\\ 0.8069\times 1.1786^{k-2},k=2,3,\cdots \end{array}$
50	$\begin{array}{l} {\hat{x}}_{1}^{\left(0\right)}(k)=0.0238\sum_{t=1}^{k-2}\left[\sum_{i=2}^{4}1.0236^{t-1}b_{i}x_{i}^{\left(1\right)}(k-t)\right]+\\ 1.0118\sum_{i=2}^{4}b_{i}x_{i}^{\left(1\right)}(k)+0.0128\sum_{j=0}^{k-3}1.0236^{j}+0.0236\times 1.0236^{k-2}x_{1}^{\left(0\right)}(1)+\\ 0.8620\times 1.0236^{k-2},k=2,3,\cdots \end{array}$
75	$\begin{array}{l} {\hat{x}}_{1}^{\left(0\right)}(k)=0.2613\sum_{t=1}^{k-2}\left[\sum_{i=2}^{4}1.2339^{t-1}b_{i}x_{i}^{\left(1\right)}(k-t)\right]+\\ 1.1170\sum_{i=2}^{4}b_{i}x_{i}^{\left(1\right)}(k)-0.0983\sum_{j=0}^{k-3}1.2339^{j}+0.2339\times 1.2339^{k-2}x_{1}^{\left(0\right)}(1)+\\ 0.5764\times 1.2339^{k-2},k=2,3,\cdots \end{array}$

**Table 10:** Model parameters and accuracy test.

Number of Seawater Freeze–Thaw Couplings	Strength Loss Rate Prediction Model		Relative Dynamic Elastic Modulus Prediction Model	
	a	Average Relative Error	a	Average Relative Error
25	−0.1733	4.9498%	−0.1639	0.3806%
50	−0.0366	4.7651%	−0.0233	0.9743%
75	−0.2125	3.4281%	−0.2094	0.9890%

## Data Availability

Data are contained within the article.
